# Glycogene Expression Profiling of Hepatic Cells by RNA-Seq Analysis for Glyco-Biomarker Identification

**DOI:** 10.3389/fonc.2020.01224

**Published:** 2020-07-28

**Authors:** Kiyohiko Angata, Hiromichi Sawaki, Shigeko Tsujikawa, Makoto Ocho, Akira Togayachi, Hisashi Narimatsu

**Affiliations:** Molecular and Cellular Glycoproteomics Research Group, Department of Life Science and Biotechnology, Cellular and Molecular Biotechnology Research Institute, National Institute of Advanced Industrial Science and Technology, Tsukuba, Japan

**Keywords:** glycosyltransferase, next-generation sequencing, quantitative real-time-PCR, biomarker, transcriptome

## Abstract

Glycans are primarily generated by “glycogenes,” which consist of more than 200 genes for glycosynthesis, including sugar-nucleotide synthases, sugar-nucleotide transporters, and glycosyltransferases. Measuring the expression level of glycogenes is one of the approaches to analyze the glycomes of particular biological and clinical samples. To develop an effective strategy for identifying the glycosylated biomarkers, we performed transcriptome analyses using quantitative real-time polymerase chain reaction (qRT-PCR) arrays and RNA sequencing (RNA-Seq). First, we measured and analyzed the transcriptome from the primary culture of human liver cells and hepatocarcinoma cells using RNA-Seq. This analysis revealed similar but distinctive expression profiles of glycogenes among hepatic cells as indicated by the qRT-PCR arrays, which determined a copy number of 186 glycogenes. Both data sets indicated that altered expression of glycosyltransferases affect the glycosylation of particular glycoproteins, which is consistent with the mass analysis data. Moreover, RNA-Seq analysis can uncover mutations in glycogenes and search differently expressed genes out of more than 50,000 distinct human gene transcripts including candidate biomarkers that were previously reported for hepatocarcinoma cells. Identification of candidate glyco-biomarkers from the expression profile of the glycogenes and proteins from liver cancer tissues available from public database emphasized the possibility that even though the expression level of biomarkers might not be altered, the expression of the glycogenes modifying biomarkers, generating glyco-biomarkers, might be different. Pathway analysis revealed that ~20% of the glycogenes exhibited different expression levels in normal and cancer cells. Thus, transcriptome analyses using both qRT-PCR array and RNA-Seq in combination with glycome and glycoproteome analyses can be advantageous to identify “glyco-biomarkers” by reinforcing information at the expression levels of both glycogenes and proteins.

## Introduction

Expression of glycoproteins on the cell surface and into extra cellular space depends on the cellular conditions that are evidenced by the altered expression profiles of glycoproteins in conditions such as acute or chronic diseases and cancers, as compared to normal conditions (1–4). We recently succeeded in identifying glyco-biomarkers in liver fibrosis and cholangiocarcinoma by the systematic identification of these glycoproteins (5–9). Importantly, the expression of glycoproteins can be determined by analyzing the glycogenes, including glycosyltransferases, nucleotide-sugar transporters, and glycosidases as well as certain proteins that act as glycan acceptors. Evaluating the expression levels of glycogenes is a useful step in studying the biological phenomena involving biomarkers. So far, more than 200 genes have been listed as glycogenes (10) and can be partly found in the GlycoGene DataBase (GGDB, https://acgg.asia/db/ggdb/) (11). Currently, there are multiple gene expression profiling techniques such as DNA microarray that can be used to analyze genome-wide gene expression, although it is still difficult to detect weakly expressed genes (12–14). Since the expression levels of several glycogenes are generally low, some of them were barely detectable in our DNA microarray analysis. Thus, it was necessary to obtain accurate quantification and comprehensive expression profiling of as many glycogenes involved in the glycan synthesis pathway as possible.

Quantitative PCR (qPCR) assay is considered as the most reliable method for studying gene expression due to its high accuracy and detection sensitivity (14). As it is generally time-consuming to perform qPCR analysis for multiple assays, improvements have been made for enhancing the capacity and multiplicity of the qPCR method (15–17). Recent digital PCR applications are sensitive enough to analyze the expression of low expressing glycogenes. Although these techniques can be effectively used for high-throughput analysis, it is still hard to prepare a set of target genes of interest. In fact, many glycogenes are expressed at relatively low levels and are not listed in commercially available assays. Thus, we designed a one-shot expression assay for 186 genes using a multi-parallel qPCR array that can be performed using existing devices.

On the other hand, recent innovations in next-generation sequencing (NGS) allow us to analyze the transcriptome of particular cells and tissues (18–22). As described previously, it is crucial to identify the glycoproteins that are expressed differently in the disease cells with aberrant glycosylation than the normal cells for glyco-biomarker discovery (5, 23). Further, we need to test if the NGS-based transcriptome analysis can be applied to measure the expression levels of glycogenes to uncover differences in the expression of glycoproteins between normal and cancer cells.

In the present study, we report a quantitative analysis using a quantitative real-time PCR (qRT-PCR) array for evaluating the expression levels of 186 glycogenes in normal and cancerous hepatic cells. We also measured glycogene expression of the same cell lines using RNA sequencing (RNA-Seq) along with NGS. Comparative study revealed that the transcriptome analysis is reliable enough to obtain the glycogene expression profiles to identify the candidate glycoproteins that are differentially expressed in cell lines. Thus, we propose that introducing both a qRT-PCR array and RNA-Seq analysis into our experimental strategy will promote the identification of glyco-biomarkers.

## Materials and Methods

### Cell Culture

Primary human hepatic (PHH) cells were purchased and cultured in hepatocyte culture media (LONZA, Walkersville) at 37°C with 5% CO_2_ according to the manufacturer's recommendations. The hepatocarcinoma cell (HCC) lines, HepG2, HuH7, HLF, and PLC/PRF/5 and the control cell line HEK293 obtained from ATCC or JCRB cell bank were cultured in Dulbecco's modified Eagle's medium or RPMI1640 medium supplemented with antibiotics and 10% fetal bovine serum. HAK1A and HAK1B (24) kindly gifted HCC lines were cultured in RPMI1640 medium with antibiotics and 10% fetal bovine serum.

### qRT-PCR Array

To establish the qRT-PCR array system, we first generated the reference plasmids containing 186 glycogenes and three control genes. The nucleotide sequences of the target genes were obtained from the RefSeq collection on the National Center for Biotechnology Information website. Primer pairs and probe sequences used for the arrays were designed using the ProbeFinder software (https://www.roche-applied-science.com/) and synthesized by FASMAC (Japan). The template cDNAs for PCR were either obtained from the glycogene library at our institute [AIST, Japan (25)] or were purchased from Open Biosystems (Huntsville, AL). After sequencing the target regions to be amplified, the quality and quantity of the cloned plasmid DNAs were confirmed using a photometer and agarose gel electrophoresis. Each plasmid DNA was linearized using the appropriate restriction enzymes, and the concentration of each DNA was adjusted to 16 nM, which was used as the reference template in the qRT-PCR assay.

Next, we performed qRT-PCR arrays using 384-well-plates with a programmable dispenser (MLS-96; Nikkyo Technos, Tokyo, Japan), which was equipped with 96 cylindrical heads and three chilled stages. Following that, all 189 primer pairs and probes were placed in a pair of 96-well-reservoirs and dispensed into the 384-well-plates in duplicates. The assay plates were dried, sealed individually, and stored in plastic shaded bags with a desiccant in a cool and dark place until further use. The reaction mixture, including qPCR Quick GoldStar Mastermix Plus (Eurogentec) and templates, was dispensed into the assay plate on the chilled stage with the concentration of each PCR primer or hydrolysis probe was set at 0.1 μM. A LightCycler 480 (Roche Diagnostics) was used to measure the concentration of each target DNA under optimal cycling conditions (50°C for 2 min, 95°C for 2 min, and 55 cycles of 95°C for 15 s and 60°C for 1 min 20 s). The calibration curves for the individual assay sets were generated from triplicate measurements using reference plasmid templates, which contained 105 or 103 copies of the template cDNAs as described above.

The hepatic cells were cultured and the total RNA was extracted using a RNeasy plus mini kit (QIAGEN) according to the manufacturer's instructions. First-strand cDNA was synthesized with a QuantiTect Reverse cDNA Transcription kit (QIAGEN) using 4 μg of total RNA and the serially diluted cDNAs were subjected to the qRT-PCR array as described above by replacing the templates with the cDNAs. The measured values of the crossing points (Cp) were determined by the second derivative max method (26). The normalization procedure was similar to the average-scaling method commonly used for DNA microarray analysis.

### RNA-Seq Using NGS

The total RNA from the same cell lines was prepared using an RNeasy Plus Mini kit as described above. The mRNA from each cell line was further purified with a Dynabeads mRNA DIRECT Micro Kit (Thermo Fisher Scientific). To prepare the cDNA libraries for NGS, an Ion Total RNA-Seq Kit (Thermo Fisher Scientific) was used to digest the mRNAs using RNase and to amplify the fragmented DNA after cDNA synthesis according to the manufacturer's instructions. The RNA or DNA concentration was measured using the Agilent Bioanalyzer and the cDNAs were further amplified on Ion Sphere Particles using emulsion PCR with Ion One Touch 2 and enriched using One Touch ES (Thermo Fisher Scientific).

The cDNA library on the Ion Sphere Particles was applied to an Ion 318 chip and the sequencing reaction was carried out using an Ion PGM Sequencing 200 Kit (Thermo Fisher Scientific). The sequences obtained from the Ion PGM were collected and analyzed using a CLC Genomics Workbench (Qiagen). We repeated both the RNA preparation and NGS run twice from each cDNA library preparation, resulting in four cycles of NGS and about 10 million reads per cell line to perform statistical analyses of differential gene expression. The data output in the fastq file format contained information about sequences and quality (Phred quality score) and the sequences with average Phred scores higher than 20 per position were used for alignment. All the sequenced data have been deposited in DDBJ database (BioProjet Accession: PRJDB9068, DRA Accession: DRA010254 to DRA010261).

### Comparative Analysis of RNA-Seq and Microarray Data

To confirm the quantitative accuracy of the gene expression data obtained by RNA-Seq, we compared the RNA-Seq data with the transcriptome data analyzed using DNA microarrays. The transcriptome data, GSM618131 registered to the Gene Expression Omnibus (GEO) database (http://www.ncbi.nlm.nih.gov/geo/), were used as raw data of HuH7 cells based on the DNA microarray analysis (27). The data were normalized with R software according to the robust multi-array average (RMA) method. The transcriptome data of HuH7 cells obtained by the RNA-Seq technique were normalized and then Log2-converted, followed by the correlation analysis by using JMP11 (SAS Institute Inc.).

### Analyses of RNA-Seq Data From Hepatic Cells

The reads were mapped on the genomic DNA data from hg19 and assembled after removing low-quality reads or adaptors sequences using the CLC Genomics Workbench (Qiagen). To compare the expression level within the same cell lines, we calculated the reads per kilobase of exon model per million mapped reads (RPKM). On the other hand, the total reads were used for analyzing the differential gene expression. For comparative analysis of the glycogenes, only the glycogenes were extracted from the mapped sequences by using an in-house program. We matched the aligned data with the RefSeq genes for RNA-Seq to identify and quantify the directions of variability in the data. We compared the RPKM of each glycogene using Microsoft Excel and JMP13 (SAS Institute Inc.) to perform a principal component analysis (PCA) and cluster analysis by Ward's method.

### Collection of RNA-Seq Data From the Cancer Genome Atlas (TCGA)

We used the RNA-Seq data generated by the TCGA Research Network (https://www.cancer.gov/tcga) to analyze the expression levels of the glycogenes and candidate biomarker proteins. To ensure uniformity in the data quality in the Liver Hepatocellular Carcinoma project (TCGA-LIHC, 377 cases), we selected TCGA-DD and retrieved 32 of 151 cases (summarized in Table S3).

### Analysis of Differential Gene Expression

We obtained the RefSeq gene annotation for all known genes in the human genome, hg19 using the CLC Genomics Workbench ver.12. Approximately 57,000 unique transcripts with genomic information were included in this annotation. The total reads mapped to hg19 were used to calculate the number of reads and were listed in a table with the RefSeq annotation. The obtained total read counts from RNA-Seq of PHH and HCC cells were used to calculate the fold changes and *p*-values with a false discovery rate (FDR)-correction by Empirical analysis of DGE, which implements the Exact Test used in the edgeR package, in Genomics Workbench. This generated table was further utilized to make volcano plots to present differential gene expression with *p*-values using the RNA-seq analysis tools.

### Pathway Analysis

To analyze the dynamic changes in gene expression between PHH and HCC cells, a list of genes involved in pathways determined by KEGG (https://www.genome.jp/kegg/) were retrieved using Gene Set Enrichment Analysis (GSEA). The fold changes for the selected genes calculated as shown above were extracted and expressed in a table of differentially expressed genes for each pathway. The number of genes with a fold change >2 and an FDR-corrected *p*-value lower than 0.05 was counted to compare the differences in the expression profiles among pathways.

### Statistical Analysis

As described above, we used the JMP13 and CLC Genomics Workbench for the statistical analysis of the expression profiles obtained from RNA-Seq, The implementation of the “Empirical analysis of DGE” algorithm in the Genomics Workbench generally used for the most parts the default settings in the edgeR package according to the manufacture's manual (Qiagen). An adjustment of the *p*-value was performed with an FDR calculation (28).

## Results

### Establishing qRT-PCR Array System

**Figure 1:**
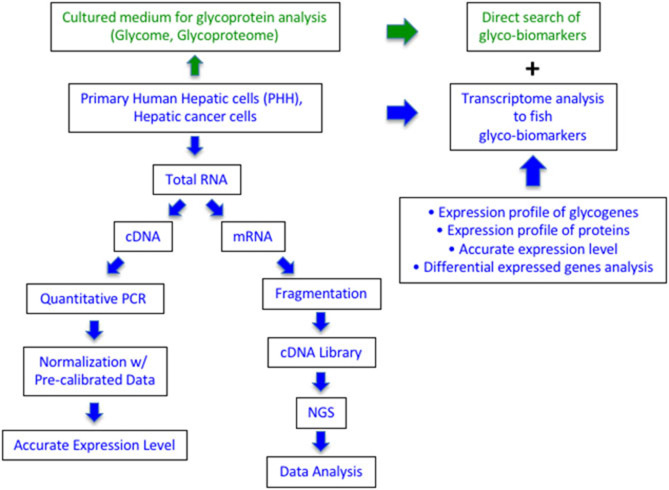
Comparison of qRT-PCR array and RNA-Seq in expression analysis of glycogenes to search for glyco-biomarker. The green part is the current glycome and glycoproteome analysis using cultured media of cancer cells. The blue part is described in this study using RNA from the cultured cells. Quantitative PCR can measure the expression level as the copy number of 186 glycogenes, while RNA-Seq can collect the expression profile of all protein genes including glycogenes.

We previously proposed that the expression profile of glycogenes is applicable for determining the glyco-biomarkers (Figure 1). During the comprehensive analysis of the expression levels of glycogenes, we aimed for precise measurements, which can enable the estimation of transcript copy numbers by a single measurement per specimen using the calculation of inter-plate calibration curves, without requiring repetitive experiments. First, we prepared an equimolar mixture of double-stranded DNAs (dsDNA) from 186 glycogenes and three control genes as reference (Table S1). Since the calculation using this mixture is a critical step for performing accurate qRT-PCR array, we measured six plates, triplicates of 105 or 103 copies, every time we prepared new plates with new reagents and primers (refer to the Methods section). Next, we selected the most suitable PCR enzyme for our system from commercially available premixed PCR enzymes. The qRT-PCR system developed by us has four advantages: (1) a single assay contains as many as 186 glycogenes in duplicates on a 384-well PCR plate; (2) automated liquid handling for accurate and precise operations during qPCR array assay; (3) use of a reference equimolar template mixture containing all target glycogenes, allowing us to evaluate inter-plate variations and standardize the calibration among the preparations; and (4) versatile hydrolysis probe library to facilitate the validation of multiple assays. Once we normalized the array plates, we could easily estimate the expression level of glycogenes as a copy number and compare the values among samples. Using this system, we successfully measured the expression of glycogenes from more than 100 cell lines including lung cancer, colon cancer, prostate cancer as well as hepatocarcinoma cells (HCC) (results other than HCC will be published elsewhere by Sawaki et al.).

### Whole Transcriptome Sequencing

As the NGS technology is already established, RNA-Seq can be used for comparative expression analysis. Since the expression level is determined using the total read number of RNA-Seq, it is possible that genes with low expression levels such as certain glycosyltransferase might not be detectable enough for accurate comparison. To avoid counting the sequences encoded in both strands in the same region, we chose an RNase-based library preparation and obtained strand-specific sequences. We prepared two different libraries per cell line and performed two RNA-Seq experiments to test the minimum requirement of the library scale. Approximately 10 million reads per cell line using two preparations were utilized to estimate the relative expression level of each gene, or in other words, an average of 400 reads out of 25,000 genes. To determine if RNA-Seq can generate an expression profile comparable to a micro array using HuH7 cells (Figure S1), the RNA-Seq read number from the HuH7 cells was log2 normalized and compared with GeneChip RMA retrieved from the GEO database. The results indicated that RNA-Seq, even from a small size library, presented an expression profile similar to that of a microarray although the detailed distribution pattern of certain genes was different.

### Comparison of Glycogene Expression in Hepatic Cells

**Figure 2:**
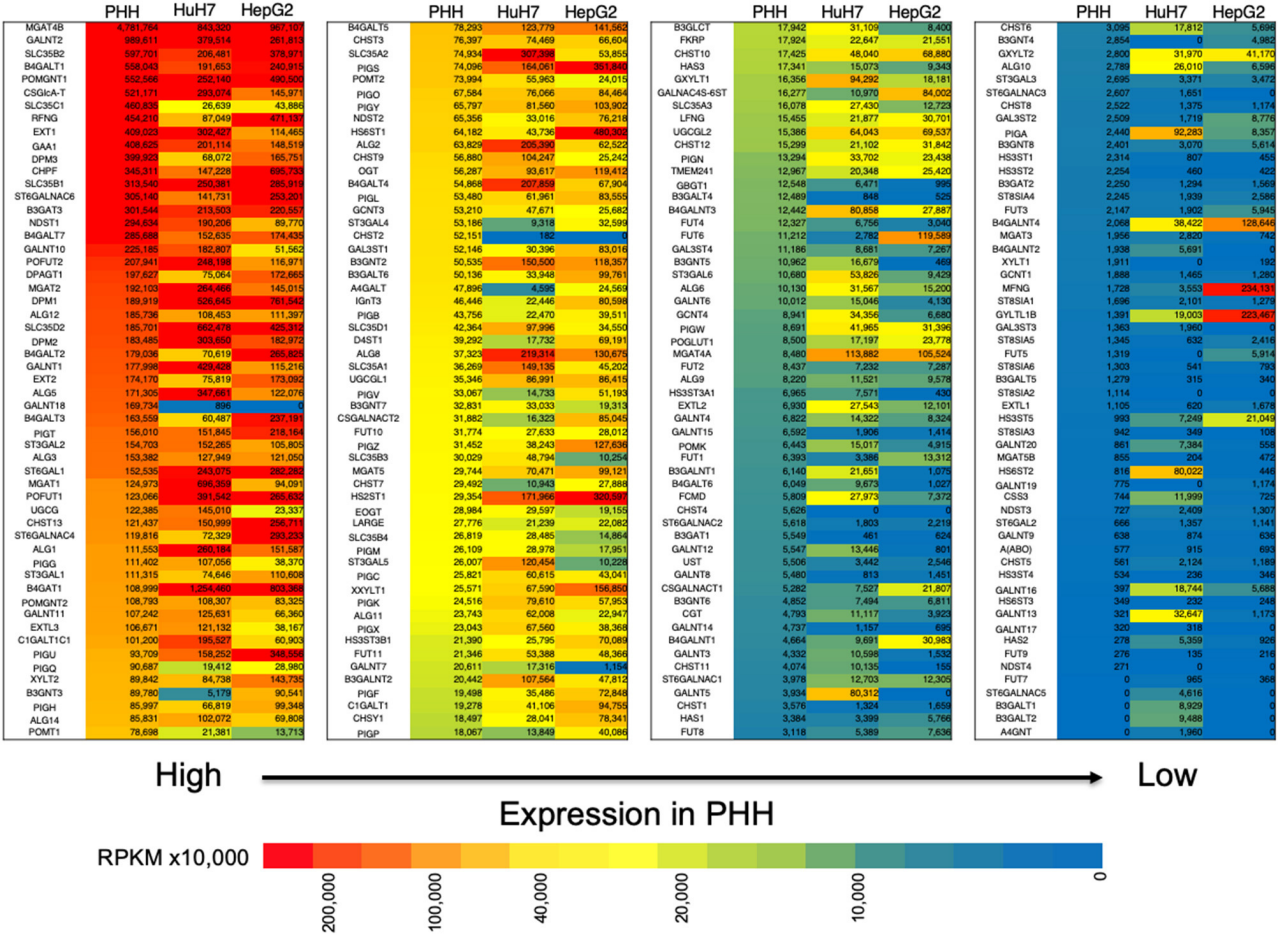
Expression of glycogenes in liver cells and hepatocarcinoma cell lines measured by RNA-Seq. The read number of the glycogenes from the RNA-Seq results of primary human hepatic (PHH) cells, HuH7 cells and HepG2 cells was used to calculate RPKM per cell line. The glycogenes are aligned from the highest to the lowest expression based on RPKM from PHH cells. Red indicates high expression levels while blue indicates low. The numbers in the figure are shown as RPKMx10,000.

In our previous study, we found that glycosylation of AFP expressed in HuH7 and HepG2 cell lines was different (29). Since glycosylation is largely affected by glycosyltransferases that are involved in glycan synthesis, we compared the expression profile of glycogenes between normal liver cells (PHH) and HCC lines (Figure 2, Table S1). In HepG2 cells, the expression of B3GNT3, FUT6, GALNAC4S-6ST, HS6ST1, and ST6GALNAC4 were higher than those in HuH7 cells. In contrast, the expression of ST3GAL5, CHST11, GALNT7, GALNT12, B3GNT5, B3GALT1, B3GALNT1, and B4GALNT2 was higher in the HuH7 cells than HepG2 cells. When the expression profile of glycogenes was compared between PHH and HCC cells, MGAT4A, HS3ST5, B4GALNT3, B4GALNT4, and GYLTL1B (LARGE2) were found to be higher in HCC while CHST2, CHST4, B3GALT4, GBGT1, B3GAT1, and GALNTL4 were higher in PHH cells. As shown in Figure 2, the heat maps representing the expression levels of each glycogene analyzed by RNA-Seq also indicate differences in the gene expression levels between PHH and HCC cells. Although qRT-PCR has a wider measurable range than RNA-Seq, similar trends were observed in the expression pattern, indicating that RNA-Seq can be useful to determine changes in the expression of glycogenes including low expressing genes.

### Differential Gene Expression Analysis

**Figure 3:**
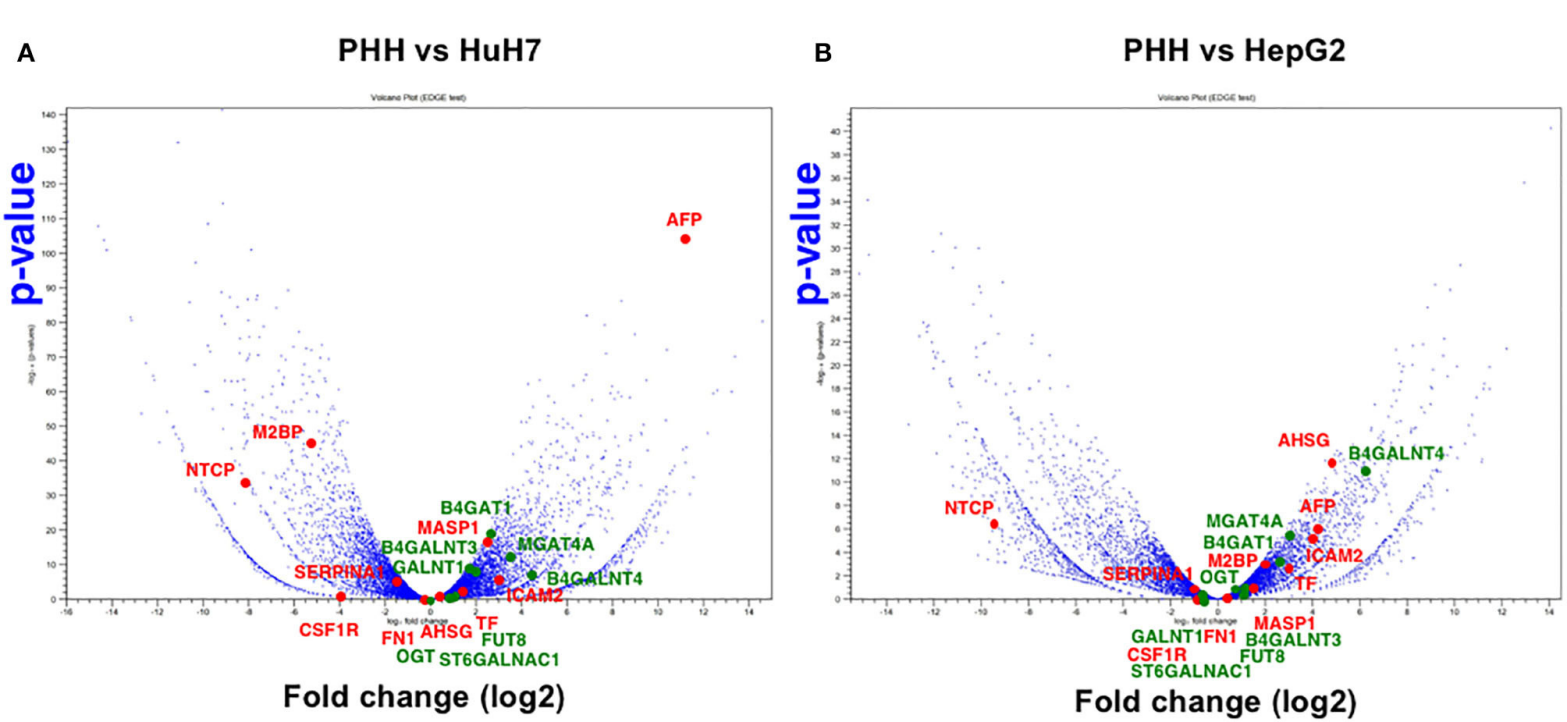
Volcano plot analysis to find differentially expressed genes in hepatic cells. Differentially expressed genes between PHH and HCC cells (**A**: PHH vs. HuH7, **B**: PHH vs. HepG2) were extracted from RNA-Seq data and the genes are plotted on significance vs. fold-change on the y- and x-axes, respectively. The y value indicates −log10 of the *p*-value obtained from the Empirical analysis of DGE. Candidates for glyco-biomarker identified in previous reports (red, 8, 29, Table 1) and glycogenes (green) are indicated as examples.

Another advantage of using NGS over a micro array is that RNA-Seq can determine the sequence information of all the expressed genes, while a micro array can assess the only measured area of targeted genes. First, we compared the expression profile of all the mapped genes. To identify the differentially expressed genes (DEGs) between PHH and the two HCC lines (HepG2 and HuH7), we subjected the RNA-Seq data sets to DEG analysis. In both cases, more than 2,000 genes showed 2-fold increase or decrease in expression level. Then, volcano plot analysis was used to find the DEGs in hepatic cells (Figure 3). For instance, SLC10A1(NTCP), a recently found receptor for hepatitis B virus (30), is expressed only in normal hepatic cells and not in HuH7 and HepG2 cells. Based on our recent report (8, 31), mass spectrometry (MS) followed by bioinformatics analysis successfully narrowed down the candidate genes for liver cirrhosis. Some of these candidate genes are also located on the volcano plots, indicating that RNA-Seq is useful for classifying the candidate genes based on their expression level. For instance, AFP, ICAM2, and MASP were highly expressed in HuH7 cells compared to PHH cells (Figure 3A). Similarly, AHSG, ICAM2, and TF were highly expressed in HepG2 cells (Figure 3B), demonstrating that RNA-Seq can support the MS data by adding information on the expression level of the candidate glycoprotein biomarkers (Table 1). As APOD, CSF1R, and PIGR were hardly detected in the RNA-Seq of HCC in the same data, we also confirmed that for RNA-Seq, cancer tissues, but not cell lines, are better sources for collecting more accurate information to find the above glyco-biomarkers (8).

**Table 1:** Expression of biomarker candidates^*^.

**Candidate gene symbol**	**Gene name**	**PHH**	**HuH7**	**HepG2**	**Fold change HuH7/PHH**	**FDR *P*-value correction**	**Fold change HepG2/PHH**	**FDR *P*-value correction**	**Stage i**	**Stage ii**	**Stage iii, iiia, iv**
A1BG	Alpha-1-B glycoprotein	35.3	6.8	70.5	−6.9	5.E-05	1.7	4.E-01	18.6	17.3	29.5
AFM	Afamin	43.8	10.3	0.0	−5.4	1.E-03	−364.1	3.E-14	66.2	55.3	72.0
AFP	Alpha fetoprotein	10.5	36568.8	235.5	2568.6	7.E-127	19.5	6.E-12	662.7	1.6	16.6
AHSG	Alpha-2-HS-glycoprotein	614.5	1490.5	15214.8	1.8	2.E-01	21.4	2.E-38	1694.7	543.2	1606.9
APOD	Apolipoprotein D	0.3	0.8	0.0	1.9	1.E+00	−3.1	1.E+00	0.9	1.6	0.7
AZGP1	Alpha-2-glycoprotein 1, zinc-binding	1453.5	0.3	531.3	−4778.0	5.E-58	−3.1	2.E-04	309.0	158.2	266.7
C1R	Omplement C1r	470.5	32.5	68.8	−18.7	8.E-13	−7.7	2.E-06	206.7	173.7	235.2
C4A	Complement C4A	2803.0	15.5	3032.3	−226.2	3.E-18	−1.1	1.E+00	N/A	N/A	N/A
C4BPA	Complement component 4 binding protein alpha	179.3	45.5	169.0	−5.2	4.E-13	−1.2	1.E+00	510.1	733.4	422.2
C4BPB	Complement component 4 binding protein beta	205.0	20.3	193.3	−13.5	2.E-19	−1.2	1.E+00	115.2	111.7	91.5
COMP	Cartilage oligomeric matrix protein	0.3	0.0	4.3	−3.3	1.E+00	10.5	1.E-02	0.3	0.4	0.7
CPB2	Carboxypeptidase B2	214.8	256.3	119.3	−1.1	1.E+00	−2.0	5.E-01	289.8	246.4	271.0
CPN2	Carboxypeptidase N subunit 2	707.5	52.3	264.3	−17.9	8.E-15	−3.0	5.E-03	67.9	88.9	90.5
CSF1R	Colony stimulating factor 1 receptor	1.8	0.3	1.0	−6.1	3.E-01	−1.9	1.E+00	6.2	5.0	5.0
F13B	Coagulation factor XIII B chain	34.3	76.3	0.3	1.7	6.E-01	−102.1	2.E-10	41.7	58.5	35.4
F5	Coagulation factor V	201.0	238.0	277.3	−1.1	1.E+00	1.2	1.E+00	79.9	104.7	80.8
FGB	Fibrinogen beta chain	4948.5	7075.3	372.0	1.1	1.E+00	−14.8	0.E+00	1727.7	2346.9	1654.8
FGG	Fibrinogen gamma chain	1060.8	2959.5	2457.3	2.1	7.E-02	2.1	1.E-02	1294.5	1713.1	1136.4
FN1	Fibronectin 1	32009.0	22277.5	45910.3	−1.9	4.E-02	1.2	1.E+00	190.1	232.8	244.8
GOLM1	Golgi membrane protein 1	524.8	76.3	3.8	−9.1	2.E-18	−151.6	9.E-44	8.0	30.8	13.6
HRG	Histidine rich glycoprotein	423.0	17.5	0.0	−31.4	3.E-29	−3594.5	5.E-44	501.7	402.5	447.9
ICAM2	Intercellular adhesion molecule 2	3.5	32.5	67.5	6.9	1.E-08	16.5	2.E-12	2.0	1.8	1.6
ICOSLG	Inducible T cell costimulator ligand	40.8	98.5	40.8	1.8	3.E-02	−1.1	1.E+00	0.1	0.2	0.2
ITIH2	Inter-alpha-trypsin inhibitor heavy chain 2	1314.3	4061.0	6631.3	2.3	3.E-03	4.4	1.E-11	827.4	526.7	741.9
LAMP2	Lysosomal associated membrane protein 2	409.0	307.3	281.3	−1.8	4.E-02	−1.6	1.E-01	50.9	37.9	43.3
LGALS3BP	Galectin 3 binding protein	554.0	18.3	2436.0	−41.6	1.E-34	3.7	6.E-05	150.1	144.9	96.9
MASP1	Mannan binding lectin serine peptidase 1	92.0	973.3	274.8	8.0	9.E-14	2.6	8.E-03	7.7	5.2	11.6
NRP1	Neuropilin 1	111.5	488.8	0.5	3.4	3.E-06	−197.3	4.E-26	7.8	8.0	9.2
ORM2	Orosomucoid 2	743.5	90.3	898.8	−11.1	3.E-07	1.1	1.E+00	1091.6	903.6	1273.9
PGLYRP2	Peptidoglycan recognition protein 2	8.0	1.5	4.5	−6.6	1.E-03	−2.1	5.E-01	46.5	27.9	55.5
PIGR	Polymeric immunoglobulin receptor	37.8	0.5	0.0	−76.2	2.E-08	−323.5	1.E-09	25.2	44.4	23.2
PTGDS	Prostaglandin D2 synthase	0.5	0.5	12.5	−1.2	1.E+00	17.8	3.E-07	38.2	996.0	9.4
SERPINA1	Serpin family A member 1	30859.3	11748.8	19181.8	−3.5	4.E-07	−1.8	1.E-01	4223.5	3457.2	3750.0
SERPINA3	Serpin family A member 3	2522.0	27.5	2638.5	−117.7	9.E-15	−1.1	1.E+00	3.2	4.1	3.3
SERPINA7	Serpin family A member 7	43.3	5.8	406.8	−9.8	7.E-08	8.4	6.E-09	75.7	69.6	77.1
SHBG	Sex hormone binding globulin	9.5	6.3	9.5	−2.0	3.E-01	−1.1	1.E+00	17.3	9.2	9.7
SLC10A1	Solute carrier family 10 member 1	66.5	0.3	0.0	−224.1	2.E-11	−562.0	8.E-11	57.1	59.4	59.8
TF	Transferrin	1064.5	6754.5	9728.0	4.8	8.E-03	8.1	5.E-04	755.6	540.9	857.8

* *Expression level is indicted by the average of RPKM or FPKM, N/A, not available*.

*In the fold change column, black and red indicate increase and decrease, respectively*.

### Changes in the Expression Levels of Glycosyltransferases

**Figure 4:**
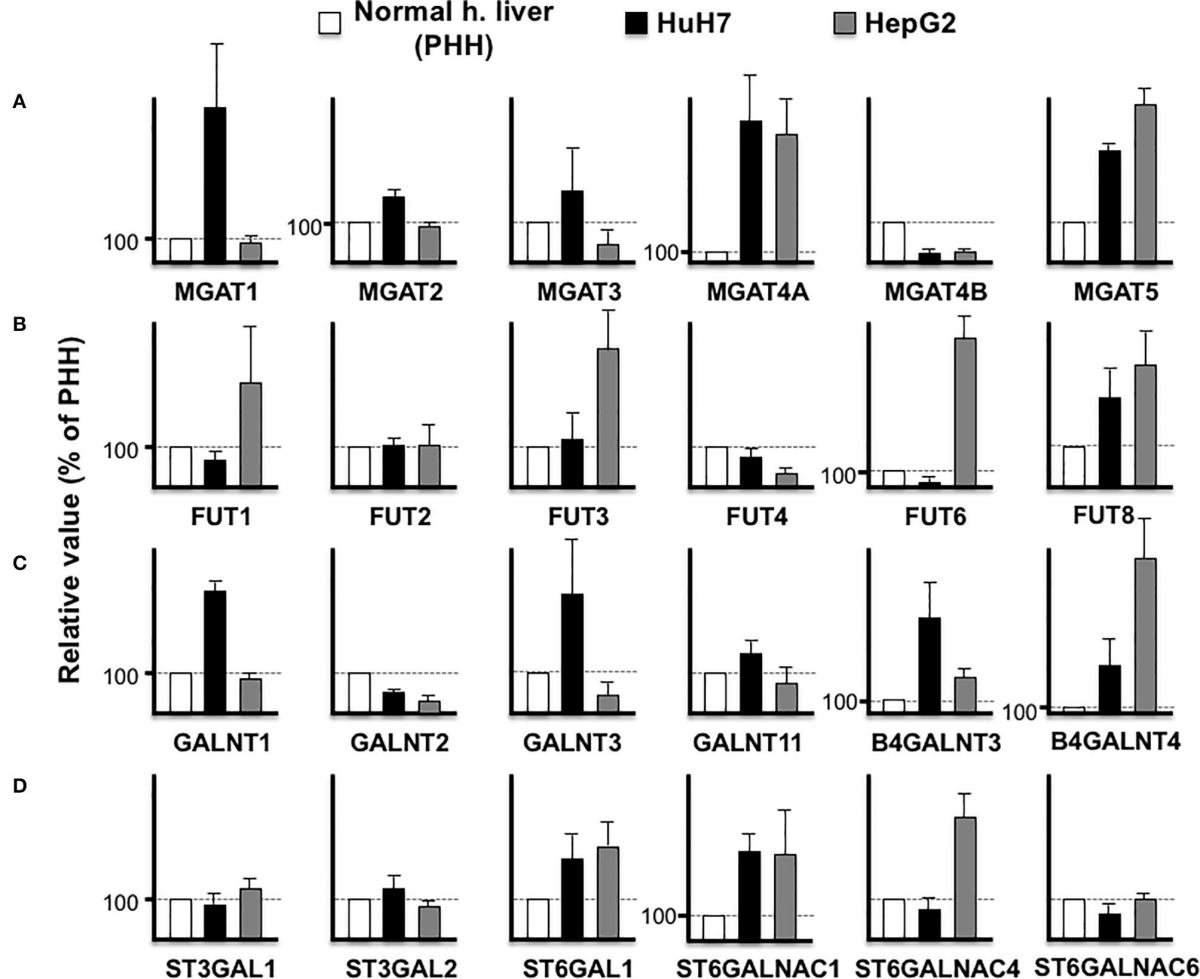
Comparison of glycosyltransferase expression among hepatic cells. RPKM obtained from PHH (white), HuH7 (black), and HepG2 (gray) were compared in N-acetylglucosaminyltransferases **(A)**, fucosyltransferases **(B)**, N-acetylgalactosaminyltransferases **(C)**, and sialyltransferases **(D)**. Average of four measurements is indicated and error bars shows standard deviations.

The aforementioned results demonstrated that an increase in the biomarker protein expression could not be observed in all the cancer cell lines, which is a major difficulty in biomarker discovery. For instance, the expression of LGALS3BP (M2BP) is lower in HuH7 than PHH cells, which makes it unsuitable as an HCC biomarker. However, as the expression level of LGALS3BP is detectable enough from the culture medium and/or serum, this protein is considered as a good indicator of altered glycosylation. The function of LGALS3BP (M2BP) as a glyco-biomarker can be attributed to Wisteria floribunda agglutin (WFA) reactivity (7), which can bind to LacdiNAc and LacNAc (32). In the HuH7 and HepG2 cell lines, B4GALNT3 is high in HuH7 cells while B4GALNT4 is high in HepG2 cells (Figure 4), suggesting that LacdiNAc can be synthesized on LGALS3BP secreted from both cells. As previously reported (29, 31), fucosylation of HepG2 and HuH7 cells is different and the expression profile of fucosyltransferases also supported the fact that glycoproteins from the HepG2 cells had higher reactivity to fucose-binding *Aleuria aurantia* lectin (AAL) than glycoproteins from HuH7 cells. Both cells express higher FUT8, which can synthesize AFP-L3, in contrast to PHH cells (Figures 3, 4).

### Changes in the Glycosylation Pathway in Cancer

**Figure 5:**
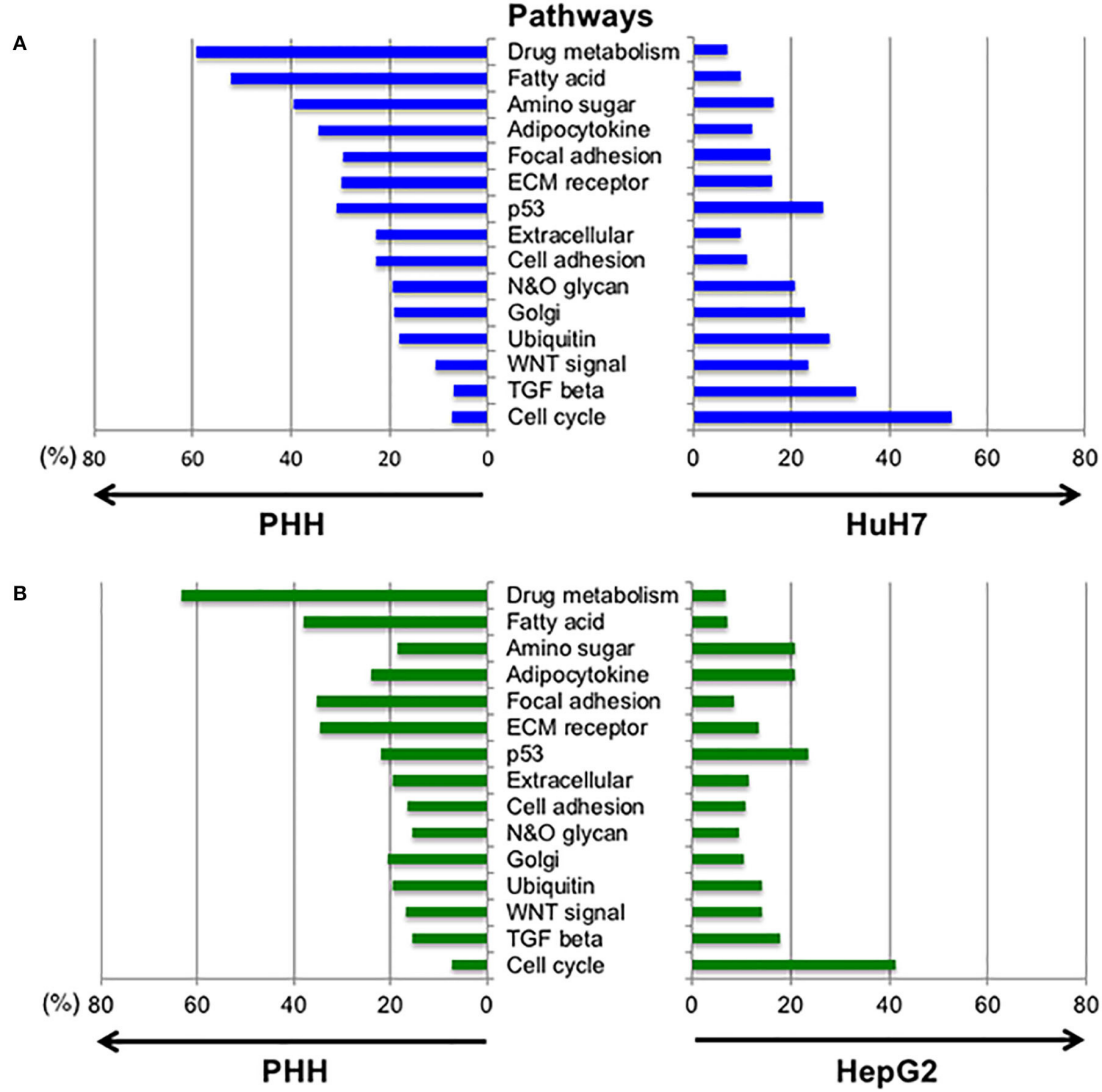
Altered expression profiles between normal and hepatic cancer cell lines in the selected pathways. To examine the changes in gene expression between PHH and HCC cells, the expression profiles of genes in different pathways were compared. Expression levels of the genes listed in 15 pathways designated by the KEGG database were extracted and the genes with fold-changes >2 and an FDR-corrected *p*-value lower than 0.05 were selected. The highly expressed genes in PHH cells compared to HuH7 cells **(A)** and HepG2 cells **(B)** are denoted at the left side of each panel while the right side shows increased expression in HCC cell lines.

Next, we analyzed the differences in the expression profiles of the pathways between the PHH and HCC cells (HepG2 and HuH7, respectively). The pathways including hepatic cell activity, cell adhesion, cell growth, membrane proteins, and extra cellular proteins were selected from the KEGG database (Figure 5, Table S2). The pathways for cell cycle, TGF-β and Wnt signaling were found to be higher in the HuH7 cell line, while metabolic pathways for fatty acids and drug metabolism were higher in PHH cells. This analysis also demonstrated that there were simultaneous alterations in ~20% of the glycogenes in the pathways for N-glycan and O-glycan synthesis in cancer cell lines, resulting in altered glycosylation of the biomarker proteins.

### Comparison of Glycogene Expression in Cancer Tissue

**Figure 6:**
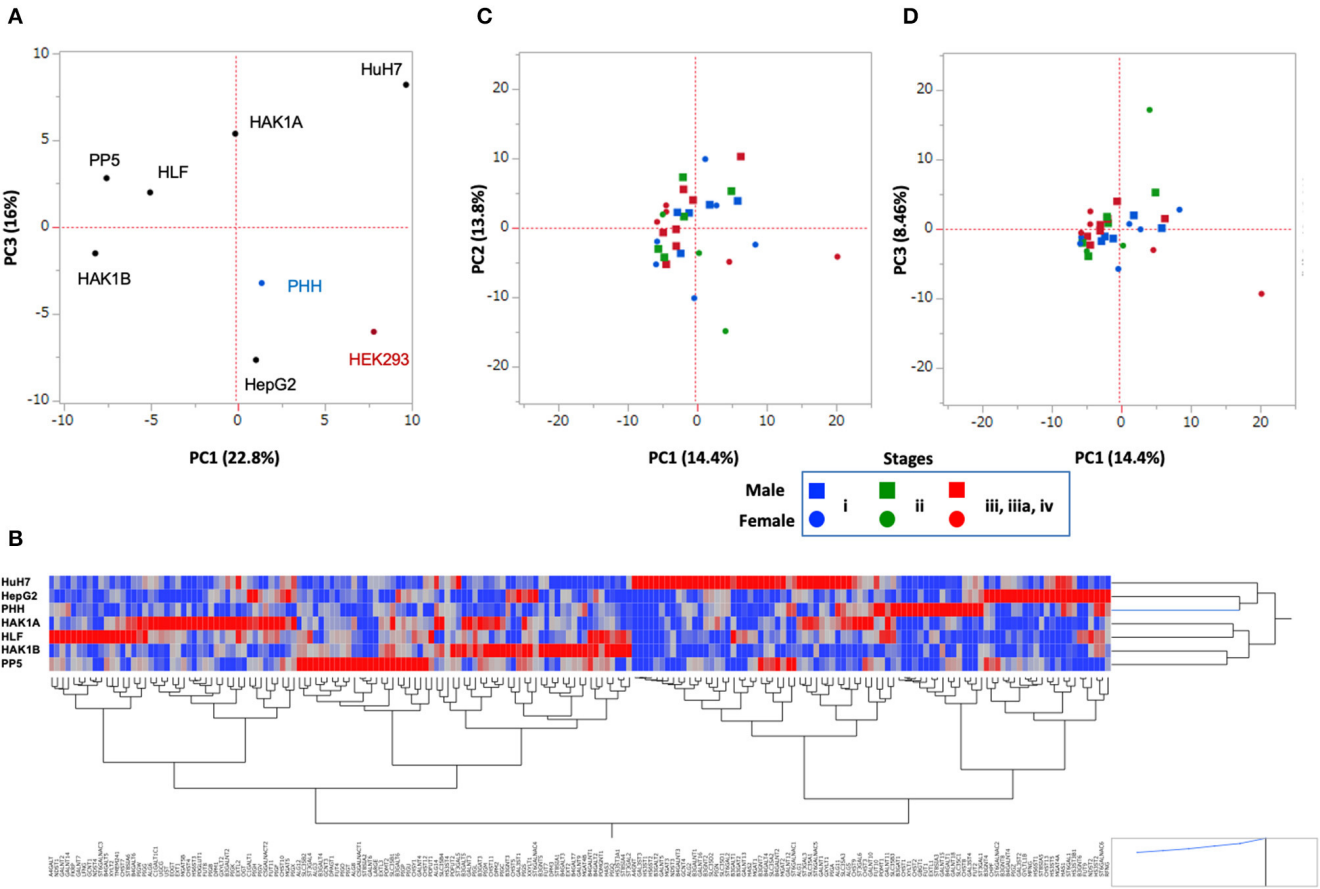
Principal component analysis (PCA) of hepatic cancer cells. Data set of the expression of glycogenes from HCC cell lines **(A,B)** and HCC tissues from TCGA **(C,D)**. **(A)** Six HCC cell lines shown in the plot were analyzed. PHH and HEK293 were used as control cells. PC1 vs. PC3 is shown. **(B)** Cluster analysis of glycogenes expression from hepatic cells. Heat map of glycogenes expression is displayed as red (strong) to blue (weak), and gene symbols are indicated under a hierarchical clustering tree. PHH, HepG2, and HuH7 are closer in this analysis. **(C,D)** The expression of glycogenes obtained from 31 patients in the TCGA-LIHC project was analyzed and plotted. PC1 vs. PC2 is in **(C)**, and PC1 vs. PC3 is in **(D)**. Note: scale is different from A. Blue: Stage i, Green: Stage ii, Red: Stage iii, iiia, and iv; Square: male, Circle: female.

The above results indicated that changes in glycosylation, which were caused by the alteration of the expression of glycogenes, took place in cancer cells. To examine how the glycosylation changed in HCC cell lines, we performed RNA-Seq of PLC/PRF/5, HLF, HAK1A, and HAK1B (Table S3), and the PCA was carried out using the expression profile of glycogenes. HCC cell lines were clearly separated from PHH and HEK293 cells as a control, suggesting HCC cell lines, except HepG2 cells, have similar changes in the expression of glycogenes. Since HepG2 is only on the same side with PHH and HEK293 cells, glycosylation in HepG2 might not be typical in HCC cell lines (Figure 6A). Furthermore, expression profiles of each cell line were analyzed by clustering analysis using Ward's method, which can display genes closely associated (red color in the heat map of Figure 6B). Consistent with the PCA analysis, the cluster analysis showed that HepG2 is the closest to PHH cells compared to other HCC cell lines (Figure 6B). Next, to evaluate the expression profile of the glycogenes in hepatic cancer tissues, RNA-Seq data of HCC were collected from The Cancer Genome Atlas (TCGA, https://www.cancer.gov/tcga) (33). Comparison of the expression of glycogenes in HCC indicated that the expression of ST3GAL1, ST6GAL1, and ST6GALNAC6 was relatively higher than other sialyltransferases (Table S4). Similarly, MGAT1 and MGAT4B were highly expressed in HCC, but MGAT4A and fucosyltransferases except POFUT1, were moderately expressed. The PCA demonstrated that the picked 31 HCC were nearly in the same area as expected (Figures 6C,D), and the distribution of stages i to iv was not observed by cluster analysis (Figure S2), suggesting that alteration of the expression of glycogene was not stage-specific but rather HCC specific.

## Discussion

### Using RNA-Seq for Quantification of Glycogenes

We aimed to estimate glycosylation changes in cancer cells using the expression profile of glycogenes. We established a qRT-PCR array to analyze 186 basic glycogenes in a 384-well-format as qRT-PCR is the most sensitive method to quantify mRNA. Our analysis indicated that RNA-Seq could measure the expression of even low expressing glycogenes and its profile is comparable to the qRT-PCR (Table S1, Figure 2). Expression of some glycosyltransferases such as fucosyltransferases in HepG2 and HuH7 were consistent with a previous report (29), and most importantly, correlated with the increase of fucosylated glycans (29, 31). Thus, RNA-Seq can be used for the quantification of glycogenes and possibly predict alterations in glycosylation (34). One of the advantages of using NGS over a microarray is that NGS can distinguish mutated genes from normal genes. In the case of a microarray and qRT-PCR array, the results highly depend on the DNA and primers for specific genes that are spotted on the arrays. As alternative splicing has been found in many genes, it is hard to identify the splice variants using a microarray and qRT-PCR array. RNA-Seq allows us to identify RNA editing, nucleotide mutations, and expression of fused genes resulting from translocation. Furthermore, mRNAs with non-sense mutations cannot be distinguished from normal transcripts in a microarray and qRT-PCR array leading to the wrong estimation due to their false-positive signals. In fact, RNA-Seq of human colon cancer LSC confirmed a non-sense mutation in N-terminal area of C1GALT1C1 (COSMC) as we previously reported [(35), data not shown]. This suggests that RNA-Seq can avoid errors in counting transcripts with mutations, which causes no or low enzymatic activity that is consistent with the recent reports (36, 37).

### Search for Glyco-Biomarkers Using Transcriptome Analyses

**Figure 7:**
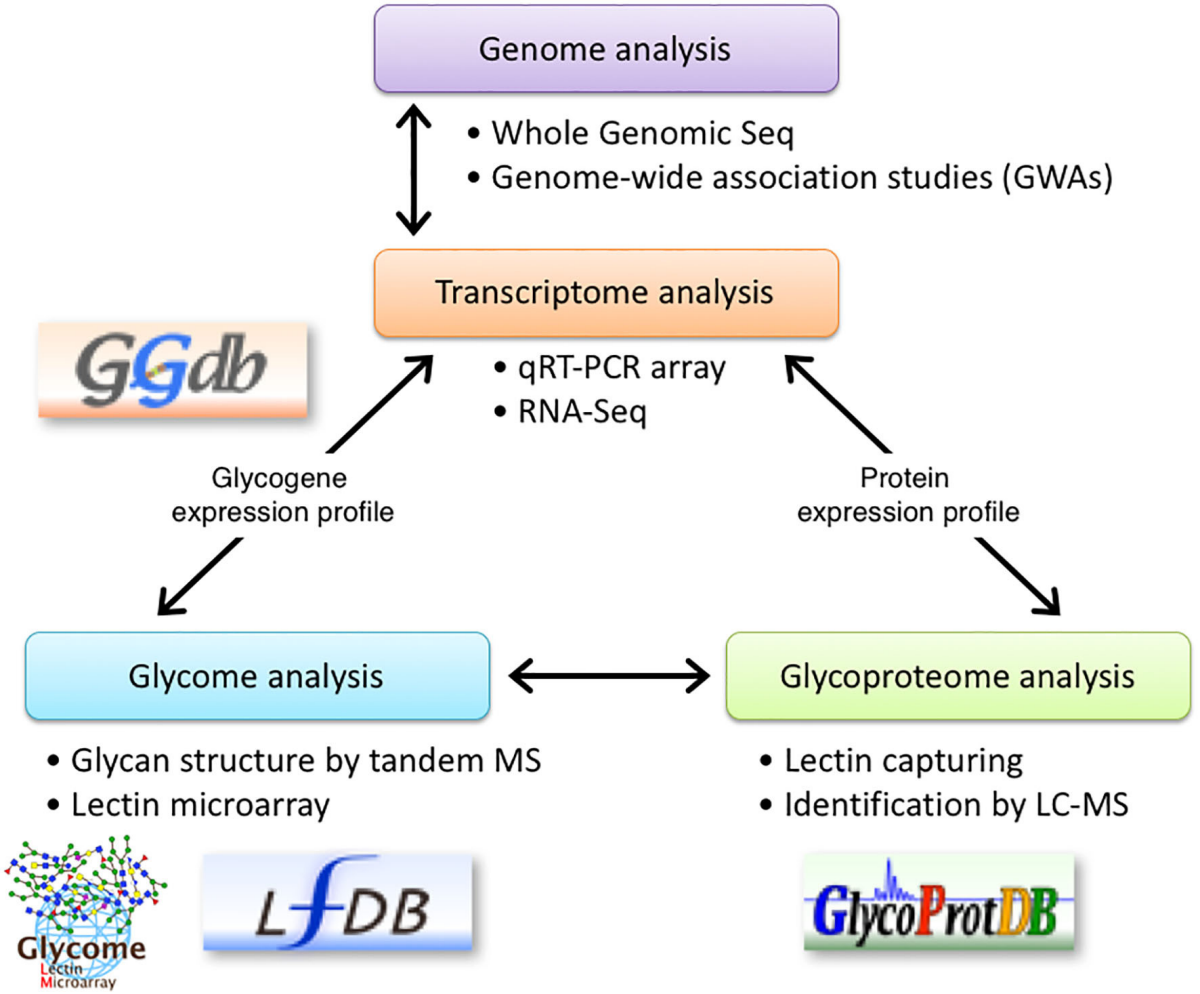
Schematic representation of glyco-biomarker identification using transcriptome analysis. RNA-seq using NGS technology is introduced into the glyco-biomarker identification strategy previously proposed by us (5). qRT-PCR array in combination with RNA-Seq will be used to determine the expression profile of glycogenes (transcriptome) and will support the glycan structure analysis by lectin microarray (glycome). Furthermore, RNA-Seq analyses will be used to determine the expression profile of candidate protein genes listed in the glycoproteome analyses. Some of these data will be stored in the indicated databases. The permissions to use logos for GGDB, LfDB, and GlycoProtDB or LM-GlycomeAtlas have been obtained either from ACGG-DB (https://acgg.asia/db/) or GlyCosmos (https://glycosmos.org).

We previously described that identifying aberrant glycosylation patterns, which differs from that in the normal cells, is the most critical step in glyco-biomarkers discovery. Since we need to detect alterations in glycans, which might occur in a minor population of glycoproteins during the early stages of cancers, we proposed to use multiple technologies for glycan analysis including a lectin microarray, tandem MS, and qRT-PCR (5). Briefly, glycan profiling of the secreted proteins using a lectin microarray helps in identifying a probe for capturing glycoproteins and for detecting biomarkers. qRT-PCR determines the expression levels of glycogenes to estimate and/or confirm alterations in the glycan structures. MS analyses can be used to identify glycan structures and sequences of the target glycopeptides, resulting in a list of potential candidates for a novel glyco-biomarker. However, in this strategy, using an antibody to detect the glycoproteins after the screening step is troublesome due to a lack of information on the expression levels of glycoproteins. Thus, we added bioinformatics to narrow down the biomarker candidates by mining the microarray database (8). Here, we added RNA-Seq in addition to qRT-PCR array for glycogenes for the identification of glyco-biomarkers (Figure 7). Although the relative expression level (= number of reads) could be affected by the expression of other genes, RNA-Seq can be used to overcome the aforementioned problems. Thus, RNA-Seq is useful to find candidate glycogenes as the first screening. It is likely that qRT-PCR collect accurate expression levels, especially low expresser genes, which reflect real biological signaling. In addition, a qRT-PCR array is convenient for the comparison of the expression level of glycogenes in multiple cell lines or tissues. Thus, using both transcriptome analysis systems, the expression levels of glycogenes and carrier molecules can be determined, which can accelerate the identification of glyco-biomarkers.

Although RNA-Seq information of cancer cell lines is necessary to characterize the glyco-biomarkers, it is important to find the best cell line representing cancer tissues. Recent bioinformatics tools and databases help in collecting basic information from clinical data such as TCGA (33, 38–40). In our analysis, we partly analyzed the TCGA-LIHC data, which has also been included in the pathology information of the human protein atlas (www.proteinatlas.org/pathology). As described above, we demonstrated that the expression of glycogenes is well-altered in hepatic cancer. B4GALT2, B4GALT3, DPM1, DPM2, HS2ST1, and PIGS are unfavorable prognostic markers in liver cancer according to the analysis by human protein atlas. Generally, glycogenes are expressed at low cancer specificity. Some of the glycogenes altered in our analysis (liver cancer) correlate with survival rate in particular cancers as shown in the same analysis. For instances, MGAT1, MGAT4A, and GXYLT2 are unfavorable prognostic markers, while MAN1C1, GCNT4, and STT3B are favorable markers in non-hepatic cancers. Protein biomarker candidates listed in Table 1 (based on 8, 30) were partly analyzed using retrieved data. As similarly shown in HCC cell lines, varied mRNA levels were detected for the candidate proteins. For example, AFP was from 0 to 3990 in FPKM and LGALS3BP (M2BP) was from 3 to 662 in FPKM. The differences in the stages were not clear in both cases. This is consistent with the interpretation of the pathology atlas of the human cancer transcriptome as non-prognostic. Because AFP-L3 and WFA-M2BP have been used as effective biomarkers, measuring only the protein expression levels is not enough to distinguish cancer cells from normal cells or diagnose the stages. Thus, these observations emphasize the importance of detecting proteins with glycosylation.

Currently, the expression level of each gene obtained by RNA-Seq is available through the human protein atlas as described above. However, it is necessary to compare the expression level among glycogenes on the way to the discovery of glyco-biomarkers. In fact, the glycogene expression profiles reflect the origin of cancer (41), suggesting the common glycosylation changes specific to each cancer type. Thus, it would be useful to open the data of the expression profile of glycogenes with their biological information, such as on the Glycogene Database (GGDB, https://acgg.asia/db/ggdb/). The data set in this manuscript will be available through the site in the near future. The difference in glycosylation can be detected by lectin microarray technology. Recently, a glycome atlas analyzed by lectin microarray (LM-GlycomeAtlas) was opened [(42), https://glycosmos.org/lm_glycomeatlas/index]. Glycan structures recognized by each lectin are available through the Lectin Frontier Database [(43), LfDB, https://acgg.asia/db/lfdb/]. If a human version of LM-GlycomeAtlas is available, glycogenes expression will be connected to glycome (glycan structures recognized by lectin). Furthermore, glycosylations on the glycoproteins are stored in the Glycoprotein Database [(44), GlycoProtDB, https://acgg.asia/db/gpdb/]. In the GlycoProtDB, glycosylation sites identified on the glycoproteins from cultured medium of HepG2 or HuH7 cells and serum of HCC patients or healthy volunteers are available as used in this analysis. Importantly, AAL recognized more glycosylation sites on many glycoproteins including FN and LGALS3BP in HepG2 and HuH7, probably due to the higher expression of fucosyltransferases in HepG2 and HuH7 than that of PHH. Similarly, many glycoproteins from HCC patients have more fucosylated sites than those from healthy volunteers as found in the GlycoProtDB. Thus, collecting the glycosylation information of serum proteins from other cancer patients using different lectins together with the glycogene expression profile will accelerate the identification of glyco-biomarkers.

In summary, we established a qRT-PCR array for glycogenes using reference DNAs to measure accurate gene expression levels and demonstrated that RNA-Seq is a powerful tool to obtain the expression profile of glycogenes together with all other genes coding for various proteins in PHH and HCC cells. Transcriptome analyses by combining qRT-PCR and RNA-Seq analyses suggested that glycan synthesis is almost regulated at the transcription level in the examined cells. In glyco-biomarker screening, comparative analyses of the transcriptomes between normal and cancer cells will facilitate the screening of candidate glycoproteins with altered glycan structures, which can be analyzed by lectin microarray (glycomic) and MS analysis (glycoproteomic) approaches.

## Data Availability Statement

The datasets generated for this study can be found in the DDBJ BioProject Accession: PRJDB9068; DRA Accession: DRA010254, DRA010255, DRA010256, DRA010257, DRA010258, DRA010259, DRA010260, and DRA010261.

## Author Contributions

KA, HS, AT, and HN conceived and designed the experiments and wrote the manuscript. KA, HS, ST, MO, AT, and HN performed experiments and the analyzed data. All of the authors contributed to manuscript revision and approved the submitted version.

## Conflict of Interest

The authors declare that the research was conducted in the absence of any commercial or financial relationships that could be construed as a potential conflict of interest.

## Supplementary Material

Figure S1 RNA-Seq data were compared using the GeneChip RMA from the GEO database obtained from HuH7 cells (GSM618131, 26). The x-axis is from the GeneChip RMA and the y-axis presents log2 transformed RNA-Seq data. The linear distribution indicates a similar expression of the gene between two different transcriptome analyses.

Figure S2 Cluster analysis of glycogenes expression from 31 patients in the TCGA-LIHC project (listed in Table S4). Cluster analysis was performed as described in “MATERIALS AND METHODS.” Patients 1–11 are stage i, patients 12–19 are stage ii, and patients 20–31 are stages iii, iiia, and iv.

Table S1 Comparison of expression profiles obtained from qRT-PCR and RNA-Seq.

Table S2a Expression difference between PHH and HuH7 cells for Figure 5A.

Table S2b Expression difference between PHH and HepG2 cells for Figure 5B.

Table S3 Expression profile of glycogenes from hepatic cells.

Table S4 Comparison of expression of glycogenes and biomarker candidates in RNA-Seq data extracted from TCGA-LIHC.
