# Supplementary figures and images for: Glycogene Expression Profiling of Hepatic Cells by RNA-Seq Analysis for Glyco-Biomarker Identification
Source: Front Oncol. 2020 Jul 28;10:1224. doi: 10.3389/fonc.2020.01224 (PMC7402167; doi:10.3389/fonc.2020.01224)

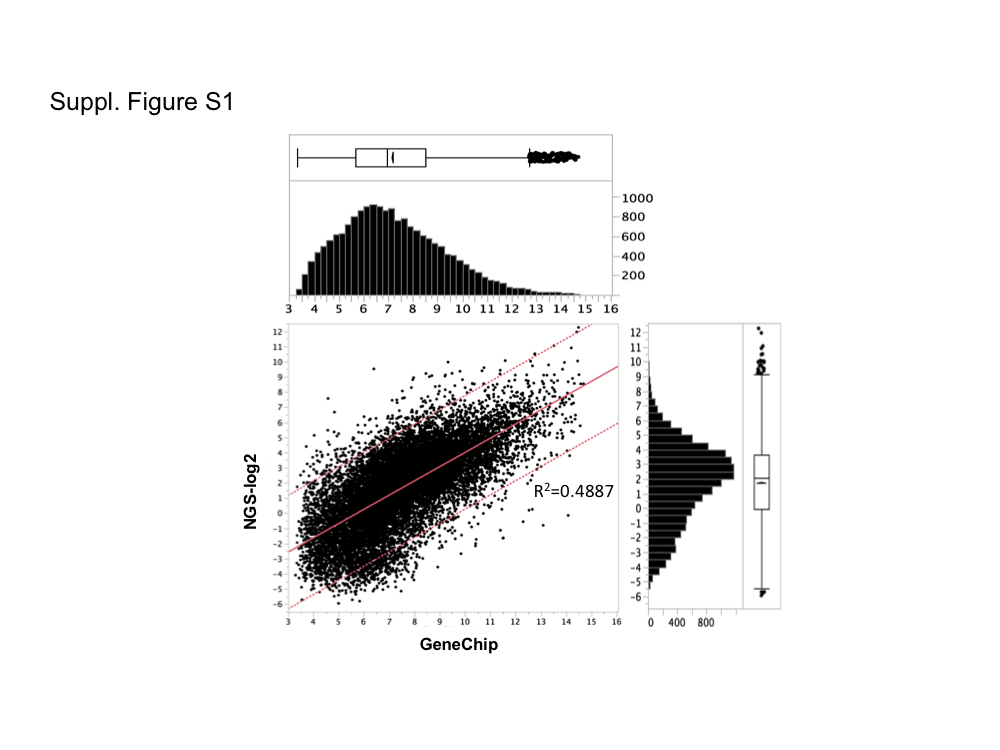

Supplement: Figure S1 — RNA-Seq data were compared using the GeneChip RMA from the GEO database obtained from HuH7 cells (GSM618131, 26). The x-axis is from the GeneChip RMA and the y-axis presents log2 transformed RNA-Seq data. The linear distribution indicates a similar expression of the gene between two different transcriptome analyses. [file Image_1.TIFF]

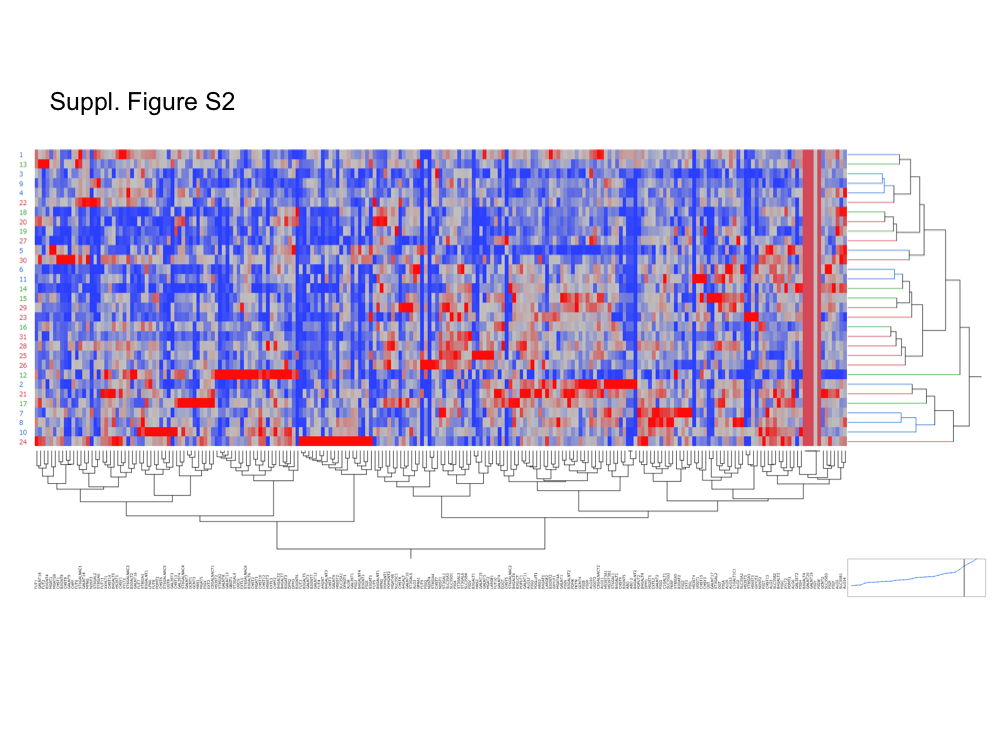

Supplement: Figure S2 — Cluster analysis of glycogenes expression from 31 patients in the TCGA-LIHC project (listed in Table S4). Cluster analysis was performed as described in “MATERIALS AND METHODS.” Patients 1–11 are stage i, patients 12–19 are stage ii, and patients 20–31 are stages iii, iiia, and iv. [file Image_2.TIFF]
